# Changes in Healthcare During the Past 30 Years: Can the National Health Service in the United Kingdom Survive?

**DOI:** 10.7759/cureus.38120

**Published:** 2023-04-25

**Authors:** Sean R Bennett

**Affiliations:** 1 Anesthesiology/Cardiac, University Hospital Southampton NHS Foundation Trust, Southampton, GBR

**Keywords:** secondary care, primary care, defensive medicine, global healthcare system, national health service

## Abstract

At the turn of the century, the National Health Service (NHS) in the United Kingdom (UK) was considered one of the top public healthcare systems in the world. Not only was it comprehensive and inclusive, but it was also free at the point of delivery for the entire UK population. It was also largely available to visitors and the families of residents that lived outside the UK. During the past 30 years, the NHS has received more and more funding both in cash terms and as a percentage of the gross national product. Despite this, the general consensus is that the NHS is delivering a poor service. The current government is facing unprecedented strike action from all areas of the workforce including doctors and nurses. This editorial asks the following questions: Where has the money gone? What has caused the current crisis? Can the current NHS model survive in today’s highly technological healthcare environment?

## Editorial

The approach to this editorial is largely a personal view based on working in the National Health Service (NHS) for over 40 years and working abroad, seeing how other publicly funded healthcare systems work. The past 30 years have included time as an NHS consultant witnessing the changes on the 'shop floor' but also many years of involvement in local and regional management. The editorial marks a few of the key moments in the NHS and how these may have influenced the ability of the NHS to meet the demands of the general public.

The emergence of the NHS in 1948

Three years after the end of the Second World War, the NHS started. It was a bold move that was initially resisted by many doctors who previously had run a private healthcare system. A service that would be free at the point of delivery would increase access and, therefore, demand for healthcare. Post-war, the country was heavily in debt. Where would the staff required for this come from? Training of doctors and nurses was stepped-up, but large numbers came from overseas, especially from Asia, to fill the gaps and provide a service. Most doctors were 'generalists' as the scope of medicine was very limited. The first antibiotics had just been introduced, surgery was basic, and there was no open-heart surgery. The predicted cost of starting the NHS was 176 million pounds sterling. This more than doubled in the first year. Doctors were paid on an annual contract, and the day's work was determined by the number of patients that needed to be seen. General practice (GP) or primary healthcare was the backbone of the NHS providing the majority of patient-doctor contacts and relatively few patients being referred to secondary or hospital healthcare. This continued for almost 50 years at which time healthcare options expanded steadily at an ever-increasing cost.

The past 30 years

As a medical student and junior doctor, one was unaware of the costs. The job was to learn medicine and become a doctor in one’s chosen field. Hours of work were loosely defined. The job revolved around getting the work done and being available for your patients. There was no European Working Time Directive (EWTD), and hospital doctors worked in close-knit teams called firms. Despite the fact that most medical schools only offered approximately one month of training in GP during the five years at medical school, approximately half of the medical school intake went into GP after a few years of working as a junior doctor. Some doctors emigrated, mostly to seek overseas experience and return to the NHS.

Many doctors took on secondary roles; for example, a GP might give anaesthetics once a week or run the local hospital. Anaesthetists would work across all specialties. Likewise, general medical physicians and general surgeons would cover all aspects of their specialty. Sub-specialty training was just starting. Intensive care was just starting. At first, these were ordinary wards that were converted into ICUs by taking out half the beds and putting in a secure oxygen and air supply. I remember on post-on-call ward rounds, a consultant from the firm refused to see patients that had gone to the ICU in the night, seeing 'no point as they would not survive'. The ICU was run by anaesthetists doing this work until 2012 when a separate college and training for intensive care started. Whether these were the 'good old days' or the 'dark days' depends a lot on your point of view.

Where has the money gone?

This approach meant that for the government, paying the bill, the service was very much on the cheap and relied on the dedication of its staff. There was an overriding attitude of doing everything as cheaply as possible without endangering the patient. Doctors used their judgment in this and were not constrained by protocols and other healthcare workers. Doctors relied much more on clinical assessment than tests and investigations. If they didn’t need an X-ray, blood test, or CT scan, it wasn’t done. Doctors would decide if it would be beneficial to the patient. Many advanced imaging techniques, such as coronary angiography, CT scanners, and echocardiography were only just starting, and MRI scanners did not exist. Between the years 2000 and 2014, the number of CT scanners in the United Kingdom (UK) doubled, but according to the Royal College of Radiologists, there were still only nine scanners per million population which was well below other countries such as Japan, the USA, and Australia. As such, the general doctors did not have to consider them or the specialist manpower required to run them. This is just one of many big spend items.

Orthopaedics was fixing broken bones, but after the pioneering work of Charnley's hip replacement, replacing other joints became routine. This option for the few patients suffering from arthritis soon became the standard for the vast number of people moving into their 70s, 80s, and beyond, years of age. A patient with a painful knee would be examined clinically and perhaps have an X-ray. Now, an MRI scan is standard.

There are many other areas highlighting the provision of a much-needed service for a few becoming the standard for many. Cardiac surgery then was principally for working-age patients and was only available in a few cities. Heart transplantation was very rare. Now, surgery is widely available with an average age of over 70 years. Highly technical advances in the catheter laboratory mean that coronary arteries can be stented from a peripheral artery and new heart valves can be inserted from a blood vessel in the leg. Both avoid the need for heart surgery and have brought benefits to many. One might think this would save money, but the techniques require high-cost equipment, and the bar has been lowered for intervention. This means that more patients and importantly much older patients are treated who may not have been surgical candidates, and they live to require more support from the NHS. Patients with an irregular heartbeat were treated with tablets, and now they often undergo procedures in the catheter laboratory, often under anaesthesia that takes four hours or more and a team of ten or more doctors and nurses. Commonly used blood thinning/anti-coagulant drugs for such patients were very cheap but not as convenient as much more expensive newer drugs which have the added cost that they cannot be easily reversed. This means a patient requiring surgery is offered the reversal at 1,500 pounds for one treatment. In every area, costs are spiralling.

Patients suffering from renal failure died. The past 30 years have seen a massive expansion in dialysis and renal transplantation. Patients live longer and require significant and continuous support across the NHS services.

There were very few oncology centres and few drugs to treat cancer. Now, most hospitals have oncology services, and patients who would not have received heart surgery, joint replacement, etc. because of an oncology diagnosis are no longer restricted in their treatment options. Cancer services consume more blood transfusion resources than any other single department. Not only are the drugs used expensive but are becoming much more expensive with bespoke drugs using monoclonal antibodies that target specific proteins in the patients.

Entire hospitals were built to cope with the demand for eye surgery, mostly for patients above retirement age. These treatment options were just starting 30 years ago.

In 2022, a single drug treatment for one patient became the most expensive drug given in the NHS at an estimated 2.8 million pounds.

For many years, spending per person was approximately 2,600 pounds across the UK. This rose sharply to 3,500 during COVID-19. By 2021, healthcare spending had risen to 11.9% of the gross domestic product (GDP) and had been steady at 9% for more than a decade previously (Table 1).

**Table 1:** Department of Health and Social Care Budget for England by year GDP: gross domestic product, NA: not available/applicable

Budget year	Annual budget (pounds sterling billions)	Total healthcare expenditure share of GDP	COVID-19 budget (pounds sterling billions)
1997	33	5.8%	na
2005	76	8.4%	na
2008	96	9.1%	na
2010	130	10%	na
2011	130	9.9%	na
2012	131	9.9%	na
2013	134	9.8%	na
2014	137	9.8%	na
2015	141	9.8%	na
2016	142	9.7%	na
2017	145	9.6%	na
2018	156	9.7%	na
2019	155	9.9%	na
2020	160	12.0%	40
2021	180	11.9%	42
2022	184	na	na
2023	182	na	na

All these factors equate to a large increase in equipment and manpower that was not envisaged at the outset. The NHS is not helped in this increase in manpower by the various Royal Colleges that are the gatekeepers to the post-graduate examinations and determine the amount and types of work that members are permitted to undertake. An example of this is how the running of ICUs has changed. Across the country 30 years ago, intensive care would have been covered by night and often by day by anaesthetists who would have been working mainly as anaesthetists and covering surgical work. This became unacceptable to the colleges who not only said that the anaesthetist who is covering the operating room cannot cover the ICU but also doctors who work in ICUs have to be specially trained via the new Faculty of Intensive Care Medicine. This led to an almost doubling of consultants required to run out-of-hours surgery and ICU. Double the workforce, limited increase in productivity. This has been happening across the NHS. The generalist is almost extinct. When I started as a consultant in the NHS, I was the 12th full-time consultant in the department, and when I left that department 20 years later, I was the 66th consultant. Some areas did do more work, but some did less. In my own sub-specialty, we had 50% more consultants to deliver the service but actually did 50% fewer cases. This scenario is by no means unique across the NHS with many departments carrying out fewer procedures with more manpower.

What has caused the current crisis?

Money is consumed by more equipment, more manpower, and expensive drugs. However, this is only part of the problem which can be covered by simply putting in more money.

Morale seems to be at a low ebb. This is not so easily solved with more money. There have been two key moves that caused severe blows to morale. The first was changing junior doctors'/trainees' work patterns to comply with EWTD in 2002 [1]. Countries across Europe tackled this in different ways. Those who focused on continuity of care managed it by continuing with long hours (not as long as in the 1980s) and then every 10 weeks or so a period off work to compensate. This model meant service delivery was good and trainee doctors were happy in their work. In the UK, working in firms was disbanded and trainee doctors were put on shifts similar to nursing shifts covering areas of the hospital to which they were not normally attached. The result is fragmented service delivery and crucially disenfranchised trainee doctors. One survey showed that nowadays, most consultants do not know the first names of the trainee doctors, whereas before consultants knew the names of their trainee staff, and indeed the norm was to take the firm out for dinner at least once during the period of the training job. Trainees are no longer a happy group and as I write are on strike.

Senior doctors are not affected in the same way. Here, the loss of morale and disengagement from the primary aim of the NHS seems to derive more from their loss of control over their patients and how the system is run. They remain responsible for their patients but are not responsible for some important day-to-day issues such as out-patient appointments, admissions, in-patient beds, etc. which are controlled by nurses. Tasks, such as requesting a patient to come to the operating room, have become bureaucratic and team decisions. This has resulted in slowing down the process and wasting expensive resources. Doctors are no longer in charge.

Time and motion

Consultants 20 years ago would not have tolerated many of the inefficiencies, but now they don’t want to rock the boat or be seen to cause pressure in the system. Previously, there was a common purpose to avoid cancellations, but this has gone. I worked in an operating room when a new manager decided to pay a private company to study operating room efficiency. His mission was that during working hours, operating rooms should be at least 80% efficient. The results showed that our operating rooms were over 100% efficient due to the way we all worked. The result was one of the steps toward working less. Foot on the brake, not the accelerator. This type of change was widespread. Cancellations are now routine which is wasteful and extremely stressful for the patient. I've seen some costs advertised to encourage patients not to miss appointments. These costs are low, under 100 pounds. Mostly patient attendance is excellent. I cannot imagine the cost of an entire operating room and team doing nothing because a patient is cancelled. The NHS pretends to be patient-centred but it is certainly not.

Another move that affected consultants was the new 'consultant contract' which was radically changed in 2003. This contract gave doctors more money. When they voted, they rejected it because it would change the way they would work, and it did. Work had more of a clocking-on mentality. The British Medical Association had shown that most doctors worked around two sessions above their contracted hours and most did their administrative work on their own time. This all changed. Hours were strictly time-tabled, and doctors set specific administration times, for which they were paid, but these would erode the time spent with patients. The immediate effect of this was that doctors did less clinical work for more money. However, the job became more beholden to managerial requirements and those of the General Medical Council (GMC) which took on an entirely new part of a doctor’s life following the Shipman inquiry, published in 2003 [2].

Can a change in morale have such an impact? It is difficult to be certain, but it is interesting to consider the private sector in the UK. Here, the model is very different, especially in response to the needs of the patients and clinical decisions being consultant led. The doctors have much more control, and most of their time in the hospital is spent treating patients with whom they have a rapport. Private healthcare annually accounts for around 10% of total UK healthcare. It is profit-making and does not have a staffing crisis.

The above are changes that have been occurring as a result of NHS and government policy. In addition, there have been other significant contributing factors. One was Brexit, leaving the European Union in 2021. The other was the COVID-19 pandemic around the same time. Brexit affected most industries in the UK. The NHS being the largest employer was not immune. Large numbers of workers from overseas left the country, feeling less welcome than before, and there were not enough trained UK staff to fill the roles. In addition, Brexit caused a major devaluation of the sterling, which meant the cost of importing healthcare items increased and the wages for overseas workers became less attractive. COVID-19 triggered a massive increase in spending, almost a 25% increase in NHS spending during the pandemic. What did the NHS get in return? Very little. Billions were spent on personal protective equipment that was below the NHS standard and was burnt. Billions were spent on building temporary hospitals, Nightingale hospitals, around the UK which were hardly used or not at all, and billions were spent on surveillance which most believe to have been relatively ineffective. Demonstrable wastage but for those working in busy hospitals, this wastage has had a very negative effect on morale. Now, when doctors, nurses, and others find they are being offered low-wage settlements, they feel no charity for such a wasteful government.

Defensive medicine is a major cause and cost

In 2020, a single family received a court settlement from a hospital of 37 million pounds. Recent figures show the NHS spent 3.6 billion pounds on litigation [3]. Approximately 25% goes to legal fees. Again, to those working directly with patients, this is an extraordinary amount of money. Litigation and defensive medicine are bedfellows. The more the workforce, doctors, and nurses see the reality of not only litigation but also prosecution, the more they withdraw from decision-making, that is, until decision-making becomes based on a collective or shared position that has been arrived at when all conceivable tests and risks have been explored. This may sound very reasonable, but there is a diminishing return on tests and investigations. If any aspect of the patient's history is out of place, the option to delay or cancel treatment is taken. More tests are ordered, more investigations. Individuals that try to short-circuit these processes to promote active patient management find themselves reprimanded. The, ''What if'' brigade. Productivity and cost become inversely proportional. Definitely not the model used by private hospitals. For doctors, the GMC plays a key role. Punitive action by the GMC may only affect a relatively small number of doctors, but the impact on morale and attitude is far-reaching.

GP

GP is or was the gatekeeper for the NHS and access to hospitals [4]. GPs were extremely effective in this role, and when they needed to get a patient into the hospital, the hospital doctor could assume it was genuine. This autonomy has been eroded by the same forces that are prevalent in hospitals. The lack of morale in GP has led to a manpower crisis that threatens the continuation of primary care as we know it. A primary care system that was probably the best in the world and its demise is a key factor in why secondary care cannot cope.

Waiting for definitive treatment has always been part of the deal between the public and the NHS. This is still the case. It was the fact of waiting lists that imbued an attitude of efficiency, accomplishment, and satisfaction at work. Gaps could always be filled. What upsets the public nowadays is the lack of access from the start. It is difficult to even see a GP. Hospital outpatients often are done by phone or nurses and appointments get postponed at short notice. Patients are left not knowing if they need treatment or not.

Can the current model for the NHS survive?

The NHS has always resisted overt rationing. In fact, rationing occurs all the time but by random allocation. Once in the system, the cost is no longer considered, but if you are outside waiting, even though your need may be great, you may not get the treatment required, or the window of opportunity, according to your pathology, may pass. This was quite overt during the COVID-19 pandemic [5]. Leading doctors spoke in public about patients that were dying because their treatment opportunity was missed due to late diagnosis.

Strengths and weaknesses

The relationship between the NHS and the Royal Colleges enables teaching trainees to be of a high standard, and this is recognized in the global healthcare system. However, it should not be taken for granted. Other countries also offer excellent training, and many overseas doctors find the bureaucracy involved with coming to the UK a daunting task, slow, and expensive. Some good doctors never make it. On the other hand, as many as four in 10 UK medical graduates are looking to leave the NHS. Some for their post-graduate training overseas, some permanently. This represents a massive loss to the NHS and the UK.

Certain types of research are done very well as certain conditions and regulations apply across the NHS. However multi-centre studies are done successfully in other countries. One might imagine that the NHS has a unified database for patient information. In fact, it doesn’t. National audit projects carried out by the Colleges are successful due to the work of individuals.

A vaccine for COVID-19 by AstraZeneca group was possible as a collaboration between the NHS and the private sector. The vaccine roll-out went well, as it did in many countries, but the UK mortality from COVID-19 was one of the highest in the world.

Patients who suffer from diseases requiring expensive treatments do well, for example, haemophiliacs requiring blood clotting products. However, several thousand contracted HIV in the 1980s as a result of the slow response time, to remove contaminated blood products, from the NHS.

When Charnley was developing hip prostheses and laminar flow tents, he created not only an operation of great benefit to the patient but also a new concept in clean operating room design. Unfortunately, his type of innovation and experimentation on patients may not be allowed nowadays.

The NHS always had a good pension system which was an attraction to keep a stable workforce. However, within the past 10 years, the pension has changed radically in such a way as to cause many senior doctors to retire early amplifying the workforce shortage and removing the most experienced doctors. Such was the impact that the government has now stepped in and changed the taxation system to try and reduce further losses. Unfortunately, many who have gone will not return.

The above are examples of how the NHS is not fulfilling the expectations of the general public or many of the doctors that work in it. The NHS is not surviving. The model requires an overhaul. An overhaul that involves all aspects of the health service but recognizes the fundamental importance of the doctor (hospital and GP) and patient relationship who carries responsibility; the role of nurses in their daily duty of caring for patients; the role of the GMC in supporting doctors, not acting against them in a position of 'protecting patients'; the GMC being in touch with the aspirations of school children and medical students; and Royal Colleges that remain focused on the demands of junior doctors and clinical practice. Many of the official leaders in the NHS are very comfortable and seek political correctness which will not help resolve the problems faced by the NHS.

Limitations

Throughout the text, the NHS referred to the UK. However, the UK is comprised of England, Scotland, Ireland, and Wales. Although the NHS does work in the same way across these four countries, each has its own executive which can alter service delivery to some degree. The NHS is enormous and carries out a huge amount of good work which cannot be itemized in a short article.

In conclusion, the NHS is a comprehensive, free-at-the-point-of-delivery health service. The public in the UK is proud of this service as long as high-quality healthcare is being delivered. When this starts to falter, people ask, why? It is easy to blame the current shortcomings of the NHS on the lack of funds. However, the current model, with little or no accountability as to how resources are utilized with the huge costs of modern highly technological medicine, has become unsustainable. This has been going on for several decades. The low morale across the service has been created by increased bureaucracy, defensive medicine, loss of autonomy, over-management, the relative reduction in earnings, and the breakdown of effective GP. These factors in turn have led to spiralling costs. I propose that the solutions to this need to be sought among the broad parties within and outside the NHS and that these parties need to create a clear vision as to how the NHS will cope with the ever-more technically advanced world of healthcare. The solution does not lie in only increasing funding.

## References

[1] (2023). Doctors and the European working time directive. https://www.bma.org.uk/pay-and-contracts/working-hours/european-working-time-directive-ewtd/doctors-and-the-european-working-time-directive.

[2] (2013). The Shipman Inquiry. https://www.gov.uk/government/organisations/shipman-inquiry.

[3] Lane J, Bhome R, Somani B (2021). National trends and cost of litigation in UK National Health Service (NHS): a specialty-specific analysis from the past decade. Scott Med J.

[4] Greenfield G, Foley K, Majeed A (2016). Rethinking primary care's gatekeeper role. BMJ.

[5] Richards M, Anderson M, Carter P, Ebert BL, Mossialos E (2020). The impact of the COVID-19 pandemic on cancer care. Nat Cancer.

